# Data on heteroplasmic mutations in mitochondrial genomes of loggerhead and hawksbill sea turtles: First approach

**DOI:** 10.1016/j.dib.2019.104882

**Published:** 2019-11-28

**Authors:** David Delgado-Cano, Leonardo Mariño-Ramirez, Javier Hernández-Fernández

**Affiliations:** a Department of Natural and Environmental Science, Marine Biology Program, Faculty of Science and Engineering, Genetics, Molecular Biology and Bioinformatics Research Group –GENBIMOL, Jorge Tadeo Lozano University, Cra. 4 No 22-61, Bogotá, Colombia; b NCBI, NLM, NIH Computational Biology Branch, Building 38A, Room 6S614M 8600 Rockville Pike, MSC 6075 Bethesda, MD 20894-6075, USA

**Keywords:** *Caretta caretta*, *Eretmochelys imbricata*, NGS, Heteroplasmy, Mitochondrial genome, Transcriptomes

## Abstract

The populations of loggerhead (*Caretta caretta*) and hawksbill (*Eretmochelys imbricata*) sea turtles are suffering an exponential decline due to anthropic and environmental actions that threaten their survival. In these turtle populations, the degree of heteroplasmic mutations commonly related with pathologies, has not been studied. In this data report, the specifications of each heteroplasmic site (region, mutation, length) and the percentage of heteroplasmy of each gene for four mitochondrial genomes of turtles (loggerhead: Cc1, Cc2, Cc3 and hawksbill: Ei1) are presented. The highest value of heteroplasmy in tRNA was of 83.33% for the Cc2 turtle (tRNA^Ser^ gene), in protein coding genes was 38.62% for Cc2 (ND5), and in rRNA genes of 0.74% for Ei1 turtle (rRNA-16S). The variability data obtained will be useful for further conservation projects, evolution studies and population health of these species. This is the first study of heteroplasmy in complete mitogenomes of loggerhead and hawksbill turtles.

**Table:** Specification Table

Subject Area	Biology
Specific Subject Area	Molecular biology, bioinformatics
Type of Data	Table, Text document (.fasta)
How data was acquired	*Illumina Hiseq2000*
Data format	Raw and Analyzed
Experimental Factors	Blood samples of three living loggerhead turtles and one hawksbill turtle were collected for total RNA isolates.
Experimental Features	The obtained data were assembled *de novo* with Trinity. The heteroplasmy was identified with BLASTn and multiple alignments in Geneious 6.1.6.
Data source location	CEINER Oceanarium, San Martín de Pajares Island, Cartagena, Colombia.
Data accessibility	Data of heteroplasmic sites are supplied with this article. The complete sequences of mitochondrial genomes were deposited at the GenBank database under the accession number MF554690.1, MF579504.1, MF579505.1 and MF571906.1 https://www.ncbi.nlm.nih.gov/nuccore/MF579504.1 https://www.ncbi.nlm.nih.gov/nuccore/MF579505.1 https://www.ncbi.nlm.nih.gov/nuccore/MF571906.1 https://www.ncbi.nlm.nih.gov/nuccore/MF554690.1
Related research article	M. Santibanez-Koref, H. Griffin, D. Turnbull, P. F. Chinnery, M. Herbert, G. Hudson. Assessing mitochondrial heteroplasmy using next generation sequencing: A note of caution. Mitochondrion, 46, 2019, 302–306 [10].

**Table:** 

**Value of the Data** • This is the first data report of heteroplasmy in complete mitochondrial genomes of loggerhead and hawksbills turtles. • The data reported on the position and frequency of heteroplasmic mutations can be stored in databases and subsequently compared intra and interspecifically, allowing the genetic condition of organisms to be evaluated. • The data may be relevant for researchers interested in mitochondrial mutations and their functional consequences in sea turtles. • Genetic variability data may be relevant for researchers interested in the conservation, evolution, and population health of these species.

## 1 Data description

The hawksbill turtle, *Eretmochelys imbricata* and the loggerhead turtle, *Caretta caretta*, are distributed in tropical waters and, to a lesser extent, in subtropical waters of the Atlantic, Indian and Pacific Oceans [1,2]. They are categorized by the IUCN as vulnerable species globally and in critical danger in Colombia [2] (http://www.iucnredlist.org/search). The data information of each individual is shown in Table 1. Using Hiseq 2000 platform the transcriptomes of the sea turtles were sequenced, the results are shown in Table 3.

**Table 1:** Information for each examined sea turtle individual.

Species	Abbreviation	Sex	Dimensions (cm)	Weight (kg)	Sampling date
*Caretta caretta*	Cc1	Juvenile male	57 × 62	72	April 13, 2015
*Caretta caretta*	Cc2	Juvenile female	59 × 64,5	75	April 13, 2015
*Caretta caretta*	Cc3	Adult male	81 × 92	128	April 13, 2015
*Eretmochelys imbricata*	Ei1	Juvenile female	56 × 61	69	April 13, 2015

**Table 2:** Level of heteroplasmy for each mitochondrial gene of the four individuals.

Gene	% Heteroplasmy Cc1	% Heteroplasmy Cc2	% Heteroplasmy Cc3	% Heteroplasmy Ei1
tRNA-Phe	0	0	2.9	0
rRNA-12S	0	0	0	0.2
tRNA-Val	0	0	0	0
rRNA-16S	0.18	0	0	0.74
tRNA-Leu	0	0	0	0
ND 1	0	0.1	0	0
tRNA-Ile	0	13.04	0	0
tRNA-Gln	0	0	2.82	0
tRNA-Met	0	40.58	0	0
ND 2	3.07	0.48	0	0
tRNA-Trp	1.29	0	0	0
tRNA-Ala	0	0	0	0
tRNA-Asn	0	0	0	0
tRNA-Cys	0	0	0	0
tRNA-Tyr	5.63	25.35	49.3	25.35
COX 1	2.13	0	0.71	0
tRNA-Ser	0	0	0	0
tRNA-Asp	0	0	0	37.14
COX 2	0	0	0	4.07
tRNA-Lys	0	2.74	0	65.75
ATP 8	0	0	0	28.48
ATP 6	0	0.15	0	0
COX 3	0.89	0.26	0	4.97
tRNA-Gly	14.7	1.47	16.18	0
ND 3	12.85	0	0	0.57
tRNA-Arg	68.57	0	0	7.14
ND 4L	2.31	0	0	0
ND 4	0	16	0	0
tRNA-His	0	71.43	0	7.14
tRNA-Ser	0	83.33	0	0
tRNA-Leu	0	73.61	0	0
ND 5	0	38.62	0	0
ND 6	0	0	0	0.19
tRNA-Glu	0	0	0	47.06
Cyt B	6.64	1.05	0	0.96
tRNA-Thr	9.85	1.41	0	12.68
tRNA-Pro	1.4	0	0	0
D-Loop	0	1.62	0	0

**Table 3:** Sequencing data for each individual.

Abbreviation	Number of bases (bp)	Total reads	Coverage
Cc1	5.419.023.498	53.653.698	6,3X
Cc2	4.994.908.540	49.454.540	6,1X
Cc3	5.554.620.644	54.996.244	6,6X
Ei1	5.366.738.222	53.136.022	6,3X

The importance of estimating the degree of heteroplasmy in organisms is the fact that mtDNA mutations can affect the functionality of mitochondria and generate pathologies of variable symptomatology [3,4].

The level of heteroplasmy of each gene, that is, the percentage of mutated nucleotide positions with respect to their total amount, for each turtle is shown in Table 2. The data of positions detailed changes and length of heteroplasmic mutations are shown in Supplementary Table 1 (Supplementary material).

The molecular mechanisms that cause heteroplasmy are not yet fully known, although there are five possibilities to explain the heteroplasmic variants presented here: maternal inheritance [5], introgression of the paternal genetic material [6,7], *de novo* mutations [8,9], presence of nuclear mitochondrial DNA segments (NUMTs) [10] and sequencing errors (false heteroplasmy) [11].

## 2 Experimental design, materials, and methods

### 2.1 Biological samples

Blood tissue samples from three loggerhead and one hawksbill sea turtles was obtained from the CEINER Oceanarium in San Martin de Pajares Island, Cartagena (10°11′N, 75°47′W). The blood was obtained from the dorsal cervical sinus in accordance with the Dutton [12] methodology. The samples were placed in sterilized tubes with Tris-EDTA buffer 0.1 M solution (GreinerBio-one®, Kremsmünster, Austria) and were transported at 4 °C to the Molecular Biology Lab of the Universidad Jorge Tadeo Lozano, Bogota campus. The samples were collected following the ethical standards established by the legislation with the permission from the ethics committee of the UJTL, the Ministry of the Environment for the development of the Biodiversity research (No 24 of June 22, 2012) and the Genetic Resources Access contract (No April 64, 2013). The blood samples were used for total RNA extraction using RNeasy Mini Kit (Quiagen, Hilden, Germany). For mRNA library preparation, we used a TruSeq RNA Library Prep Kit v2 according to the manufacturer's instructions (Illumina, San Diego, U.S.A.). The poly-A containing mRNAs were isolated using poly-T oligo-attached magnetic beads. The first cDNA strand followed by a second cDNA strand was synthesized from purified mRNAs. End repair was performed followed by adenylation of 3 ends. Adapters were ligated and PCR was done to selectively enrich DNA fragments with adapters and to amplify the amount of DNA in the library, respectively. The quality control of generated libraries was performed using the 2100 bioanalyzer (Agilent, Santa Clara, U.S.A.). RIN values (RNA integrity number) of 7.5 were obtained. The library was paired-end sequenced using Hiseq 2000 Platform. The quality of cleaned raw reads was verified with the fastQC program (https://www.bioinformatics.babraham.ac.uk/projects/fastqc/). Sequencing data for each individual is presented in Table 3.

### 2.2 Complete mitogenome determination

The contigs of each transcriptomes were filtered to establish the complete mitogenome for the four turtles, aligning local data obtained from sequencing against the mitochondrial genomes for hawksbill and loggerhead (access number: JX454986 and NC_016923.1, respectively) reported in GenBank (NCBI). The alignment was performed using BLASTn.

The reads of the mitochondrial sequences of each loggerhead and hawksbill turtle were saved in FASTA format. Using the Geneious 6.1.6 program (Kearse et al., 2012), all the reads for each individual were assembled using mitochondrial sequences of these turtles previously published as references (access numbers: JX454986 for hawksbill and NC_016923.1 for loggerhead turtles). For each consensus sequence, paired alignments were made with each of the 37 genes encoded by the mtDNA (reported in GenBank) using the BLAST refseq_genomic tool of the NCBI. This allowed the location of each gene within the consensus genome for each of the individuals studied.

The mitogenome sequences were published in GenBank database with the following access numbers: LMF554690.1, MF579504.1, MF579505.1 and MF571906.1 (Cc1, Cc2, Cc3 and Ei, respectively).

### 2.3 Identification of heteroplasmy

Multiple alignments were made with all the mtDNA reads of each individual and the reference mitochondrial genomes of hawksbill and loggerhead turtles reported in GenBank (access number: JX454986 and NC_016923.1, respectively), using the Geneious 6.1.6 program [13]. This procedure allowed us to obtain the reads aligned in a way that enabled a comparison among them to determine the nucleotide variations in the mtDNA of each sea turtle, discarding interindividual variations. Because the sequencing depth was low (6X), only those positions in which the frequency of the second most frequent base was greater than or equal to 30% were taken into account in order to avoid false positives. Each possible heteroplasmic site was identified: nucleotide change (wild type and mutated sequences), location (site and gene in which it is found) and length of the heteroplasmic sequence (Supplementary Table 1).

## Funding sources

This work was supported by the Office of Research, Creation and Extension of the Universidad Jorge Tadeo Lozano. Further, was funded in part the Intramural Research Program of the National Institutes of Health (USA), National Library of Medicine (USA), National Center for Biotechnology Information (NCBI) ZIA LM082713–06.
