# Supplementary material for: Data on heteroplasmic mutations in mitochondrial genomes of loggerhead and hawksbill sea turtles: First approach
Source: Data Brief. 2019 Nov 28;28:104882. doi: 10.1016/j.dib.2019.104882 (PMC6906639; doi:10.1016/j.dib.2019.104882)
Supplement: Multimedia component 1 [file mmc1.docx]

- 1. **Supplementary Table**

**Record of each mutation for each individual (coverage, nucleotide change, position and length).**

**Heteroplasmy of Ei1 turtle:**

| Heteroplasmy | Coverage on reference sequence | Nucleotide change | position | length |
| --- | --- | --- | --- | --- |
| 1 | 2/5 | A>C | (484)  rRNA-12S | 1 |
| 2 | 2/7 | G>A | (488)  rRNA-12S | 1 |
| 3 | 2/4 | TA>CT | (1526-1527)  rRNA-16S | 2 |
| 4 | 2/4 | C>A | (2705)  rRNA-16S | 1 |
| 5 | 2/5 | AC>TT | (5371-5372)  tRNA-Tyr | 2 |
| 6 | 2/5 | C>A | (5374)  tRNA-Tyr | 1 |
| 7 | 2/5 | AA>CT | (5376-5377)  tRNA-Tyr | 2 |
| 8 | 2/5 | ACACT>TGGGG | (5391-5395)  tRNA-Tyr | 5 |
| 9 | 2/5 | GCCA>TGAG | (5386-5389)  tRNA-Tyr | 4 |
| 10 | 2/5 | CCTA>AGGC | (5391-5394)  tRNA-Tyr | 4 |
| 11 | 2/4 | G>A | (7012)  tRNA-Asp | 1 |
| 12 | 2/4 | CGT>AAA | (7014-7016)  tRNA-Asp | 3 |
| 13 | 2/4 | AATTATAGGTG> CCGATCTCTAT | (7051-7060)  tRNA-Asp | 11 |
| 14 | 2/4 | A>T | (7064)  tRNA-Asp | 1 |
| 15 | 2/4 | CCCTT>GGTGA | (7066-7070)  tRNA-Asp | 5 |
| 16 | 2/4 | CGA>TTG | (7073-7075)  tRNA-Asp | 3 |
| 17 | 2/4 | T>A | (7077)  tRNA-Asp | 1 |
| 18 | 2/4 | A>C | (7079)  tRNA-Asp | 1 |
| 19 | 2/5 | A>T | (7730)  COX 2 | 1 |
| 20 | 2/5 | A>T | (7730)  COX 2 | 1 |
| 21 | 2/5 | AC>GA | (7732-7733)  COX 2 | 2 |
| 22 | 2/5 | TTGA>AGCC | (7735-7738)  COX 2 | 4 |
| 23 | 2/5 | A>C | (7740)  COX 2 | 1 |
| 24 | 2/5 | CTGA>GGCT | (7742-7745)  COX 2 | 4 |
| 25 | 2/5 | CTTC>TGCA | (7747-7750)  COX 2 | 4 |
| 26 | 2/5 | CTAA>GAGT | (7752-7755)  COX 2 | 4 |
| 27 | 2/5 | T>C | (7759)  COX 2 | 1 |
| 28 | 2/5 | TCTTAACAC> ATAGTTTGT | (7761-7769)  COX 2; tRNA-Lys | 9 |
| 29 | 2/5 | ATAGAA>TCTTGG | (7771-7776)  tRNA-Lys | 6 |
| 30 | 2/5 | C>A | (7778)  tRNA-Lys | 1 |
| 31 | 2/5 | AA>TG | (7780-7781)  tRNA-Lys | 2 |
| 32 | 2/5 | G>T | (7785)  tRNA-Lys | 1 |
| 33 | 2/5 | A>C | (7787)  tRNA-Lys | 1 |
| 34 | 2/5 | AG>GC | (7789-7790)  tRNA-Lys | 2 |
| 35 | 2/5 | GCTA>AGAG | (7792-7795)  tRNA-Lys | 4 |
| 36 | 2/5 | CCTTT>GAAAG | (7797-7801)  tRNA-Lys | 5 |
| 37 | 2/5 | A>C | (7803)  tRNA-Lys | 1 |
| 38 | 2/5 | GCTAG>AAATT | (7805-7809)  tRNA-Lys | 5 |
| 39 | 2/5 | AGAG>CCTT | (7814-7817)  tRNA-Lys | 4 |
| 40 | 2/5 | AC>TT | (7819-7820)  tRNA-Lys | 2 |
| 41 | 2/5 | CC>AA | (7822-7824)  tRNA-Lys | 2 |
| 42 | 2/5 | A>T | (7826)  tRNA-Lys | 1 |
| 43 | 2/5 | CCTCC>TGATA | (7828-7832)  tRNA-Lys | 5 |
| 44 | 2/5 | G>C | (7836)  tRNA-Lys | 1 |
| 45 | 2/5 | GA>TC | (7838-7839)  tRNA-Lys | 2 |
| 46 | 2/5 | C>A | (7846)  ATP 8 | 1 |
| 47 | 2/5 | C>A | (7855)  ATP 8 | 1 |
| 48 | 2/7 | CCT>TAA | (7876-7878)  ATP 8 | 3 |
| 49 | 2/7 | CTCTCC>AAGGAA | (7880-7885)  ATP 8 | 6 |
| 50 | 2/7 | CATG>GGAA | (7887-7890)  ATP 8 | 4 |
| 51 | 2/7 | CTAATTTA>GATTACAT | (7892-7899)  ATP 8 | 8 |
| 52 | 2/7 | CC>AA | (7902-7903)  ATP 8 | 2 |
| 53 | 2/7 | TCATCTT>AGGAAAC | (7905-7911)  ATP 8 | 7 |
| 54 | 2/7 | C>A | (7913)  ATP 8 | 1 |
| 55 | 2/7 | CCA>AAC | (7916-7918)  ATP 8 | 3 |
| 56 | 2/7 | ATTACATCTTA> CAGTATGGAGC | (7922-7932)  ATP 8 | 11 |
| 57 | 3/6 | TGGCCC> AAAAAA | (8680-8685)  COX 3 | 6 |
| 58 | 3/6 | C>A | (8687)  COX 3 | 1 |
| 59 | 2/5 | A>C | (8692)  COX 3 | 1 |
| 60 | 3/6 | CGCCT>ACTGA | (8696-8700)  COX 3 | 5 |
| 61 | 2/5 | CACAAC>ATATAG | (8705-8710)  COX 3 | 6 |
| 62 | 3/6 | TACA>TGAT | (8712-8715)  COX 3 | 4 |
| 63 | 3/6 | TG>AA | (8718-8719)  COX 3 | 2 |
| 64 | 2/5 | CAT>TGA | (8724-8726)  COX 3 | 3 |
| 65 | 2/5 | ACCATTAA> TTTGGGGG | (8728-8735)  COX 3 | 8 |
| 66 | 2/5 | G>C | (8738)  COX 3 | 1 |
| 67 | 2/5 | AAG>GCA | (8741-8743)  COX 3 | 3 |
| 68 | 2/5 | CAG>AGT | (8745-8747)  COX 3 | 3 |
| 69 | 2/5 | A>G | (9646)  ND 3 | 1 |
| 70 | 2/5 | A>T | (9665)  ND 3 | 1 |
| 71 | 2/7 | GGT>AAA | (9882-9884)  tRNA-Arg | 3 |
| 72 | 3/7 | CT>AA | (9887-9888)  tRNA-Arg | 2 |
| 73 | 2/5 | GTT>AAA | (11622-11624) tRNA-His | 3 |
| 74 | 2/4 | T>A | (11627)  tRNA-His | 1 |
| 75 | 2/4 | T>A | (11629)  tRNA-His | 1 |
| 76 | 2/7 | A>G | (14076)  ND 6 | 1 |
| 77 | 2/5 | TG>AA | (14156-14157) tRNA-Glu | 2 |
| 78 | 2/5 | TC>AC | (14161-14162) tRNA-Glu | 2 |
| 79 | 2/5 | AGACC>TCCAA | (14169-14173) tRNA-Glu | 5 |
| 80 | 2/5 | TGATTTGA>AACAAAAC | (14176-14186) tRNA-Glu | 8 |
| 81 | 2/5 | ACCG>CAAA | (14190-14193) tRNA-Glu | 4 |
| 82 | 2/5 | TGT>AAA | (14195-14197) tRNA-Glu | 3 |
| 83 | 2/5 | T>A | (14199)  tRNA-Glu | 1 |
| 84 | 2/5 | CA>GC | (14201-14202) tRNA-Glu | 2 |
| 85 | 2/5 | CT>AA | (14204-14205) tRNA-Glu | 2 |
| 86 | 2/5 | C>T | (14207)  tRNA-Glu | 1 |
| 87 | 2/5 | A>C | (14209)  tRNA-Glu | 1 |
| 88 | 2/5 | A>T | (14211)  tRNA-Glu | 1 |
| 89 | 2/5 | A>T | (14214)  Zona intergénica | 1 |
| 90 | 2/5 | GGCCA>TTTCG | (14219-14223)  Cyt B | 5 |
| 91 | 2/5 | A>C | (14225)  Cyt B | 1 |
| 92 | 2/4 | G>T | (14791)  Cyt B | 5 |
| 93 | 3/8 | TTCT>AAAA | (15362-15365) tRNA-Thr | 4 |
| 94 | 3/8 | GT>AA | (15367-15368) tRNA-Thr | 2 |
| 95 | 2/7 | GCT>AAA | (15370-15372) tRNA-Thr | 3 |
| TOTAL |  |  |  | 286 |

Heteroplasmy of Cc1 turtle:

| Heteroplasmy | Coverage on reference sequence | Nucleotide change | position | length |
| --- | --- | --- | --- | --- |
| 1 | 2/4 | C>G | (1123)  rRNA-16S | 1 |
| 2 | 2/4 | A>T | (1125)  rRNA-16S | 1 |
| 3 | 2/4 | C>T | (1127)  rRNA-16S | 1 |
| 4 | 2/5 | ACCTCAACAA>GAGATTTTGT | (4604-4613)  ND 2 | 10 |
| 5 | 2/5 | AT>TG | (4615-4616)  ND 2 | 2 |
| 6 | 2/5 | A>T | (4621)  ND 2 | 1 |
| 7 | 2/5 | T>G | (4623)  ND 2 | 1 |
| 8 | 2/5 | TAAAAC>GTTTTA | (4626-4631)  ND 2 | 6 |
| 9 | 2/5 | C>A | (4634)  ND 2 | 1 |
| 10 | 2/5 | A>T | (4636)  ND 2 | 1 |
| 11 | 2/5 | CA>AT | (4641-4642)  ND 2 | 2 |
| 12 | 2/5 | ACAAAATC>TTGTTGAG | (4644-4651)  ND 2 | 8 |
| 13 | 2/5 | A>G | (5074)  tRNA-Trp | 1 |
| 14 | 2/7 | A>C | (5370)  tRNA-Tyr | 1 |
| 15 | 2/7 | A>C | (5389)  tRNA-Tyr | 1 |
| 16 | 2/7 | CCTGTGTTTTT>AAAAAAAAAAA | (5395-5405) tRNA-Tyr; COX 1 | 11 |
| 17 | 2/7 | CCG>AAA | (5409-5411)  COX 1 | 3 |
| 18 | 2/5 | G>T | (6535)  COX 1 | 1 |
| 19 | 2/5 | G>T | (6548)  COX 1 | 1 |
| 19 | 2/5 | GCCGT>AAAAG | (6550-6554)  COX 1 | 5 |
| 20 | 2/5 | TTTGCT>AAGATA | (6556-6561)  COX 1 | 6 |
| 21 | 2/5 | T>A | (6563)  COX 1 | 1 |
| 22 | 2/5 | A>C | (6565)  COX 1 | 1 |
| 23 | 2/5 | CAGGAT>GGCTCA | (6569-6574)  COX 1 | 6 |
| 24 | 2/4 | TGAGG>AAGAT | (9453-9457)  COX 3 | 5 |
| 25 | 2/4 | TC>GG | (9459-9460)  COX 3 | 2 |
| 26 | 2/7 | GCTCCCCTAG>AAAAAAAAGA | (9463-9472) tRNA-Gly | 10 |
| 27 | 2/7 | TTAC>AGTA | (9817-9820)  ND 3 | 4 |
| 28 | 2/7 | CTCC>GGGG | (9823-9826)  ND 3 | 4 |
| 29 | 2/7 | CAC>TTA | (9830-9832)  ND 3 | 3 |
| 30 | 2/7 | G>A | (9835)  ND 3 | 1 |
| 31 | 2/7 | CCT>TGA | (9837-9839)  ND 3 | 3 |
| 32 | 2/7 | ATCTA>GGGGT | (9841-9845)  ND 3 | 5 |
| 33 | 2/7 | GAATGAGTCCAAGGAGGCTTAGAAT> TGTAATTGGGTGGGTCATATTGCTA | (9847-9871)  ND 3 | 25 |
| 34 | 2/5 | GGC>AAA | (9882-9884) tRNA-Arg | 3 |
| 35 | 2/4 | GG>AT | (9889-9890) tRNA-Arg | 2 |
| 36 | 2/4 | CTAACA>TGCTTT | (9892-9897) tRNA-Arg | 6 |
| 37 | 2/4 | A>T | (9899)  tRNA-Arg | 1 |
| 38 | 2/4 | ACTA>TAGT | (9905-9908) tRNA-Arg | 4 |
| 39 | 2/4 | TTTCGACTTAGTTAATCA> GAGTTGAGATTAAGTGGG | (9910-9927) tRNA-Arg | 18 |
| 40 | 2/5 | GATTAAACT>TCGATGTAA | (9929-9937) tRNA-Arg | 9 |
| 41 | 2/5 | C>G | (9939)  tRNA-Arg | 1 |
| 42 | 2/5 | TG>GA | (9941-9942) tRNA-Arg | 2 |
| 42 | 2/5 | TTTCCC>GCCTAT | (9949-9954) tRNA-Arg; ND 4L | 6 |
| 43 | 2/5 | AC>CT | (9954-9955)  ND 4L | 2 |
| 44 | 2/5 | CC>AT | (9957-9958)  ND 4L | 2 |
| 45 | 2/5 | T>A | (9960)  ND 4L | 1 |
| 46 | 2/5 | C>A | (9963)  ND 4L | 1 |
| 47 | 2/5 | CTTCAGC>GGCGGAG | (9965-9971)  ND 4L | 7 |
| 48 | 2/5 | ACC>GGT | (9973-9975)  ND 4L | 3 |
| 49 | 2/5 | CTCCGCC>GCTGAAG | (9977-9983)  ND 4L | 7 |
| 50 | 2/5 | T>G | (9985)  ND 4L | 1 |
| 51 | 2/5 | T>A | (9988)  ND 4L | 1 |
| 52 | 2/5 | AT>GG | (9990-9991)  ND 4L | 1 |
| 53 | 2/5 | AG>GT | (9993-9994)  ND 4L | 2 |
| 54 | 2/4 | TGGCCAC>AAAAACA | (14218-14224)  Cyt B | 7 |
| 55 | 2/4 | CT>AA | (14229-14230)  Cyt B | 2 |
| 56 | 2/5 | AT>GG | (14229-14230)  Cyt B | 1 |
| 57 | 2/5 | CGAA>ACCC | (14232-14235)  Cyt B | 4 |
| 58 | 2/5 | C>T | (14239)  Cyt B | 1 |
| 59 | 2/5 | GCTGACC>TTCTCCA | (15207-15213)  Cyt B | 7 |
| 60 | 2/5 | A>C | (15215)  Cyt B | 1 |
| 61 | 2/5 | C>T | (15222)  Cyt B | 1 |
| 62 | 2/5 | AC>TT | (15225-15226)  Cyt B | 2 |
| 63 | 2/5 | TG>CT | (15228-15229)  Cyt B | 2 |
| 64 | 2/5 | TGGAGG>ACCCAC | (15233-15238)  Cyt B | 6 |
| 65 | 2/5 | CA>TT | (15240-15241)  Cyt B | 2 |
| 66 | 2/5 | CAGTCGA>ACACATC | (15244-15250)  Cyt B | 7 |
| 67 | 2/5 | G>A | (15252)  Cyt B | 1 |
| 68 | 2/5 | T>A | (15254)  Cyt B | 1 |
| 69 | 2/5 | C>A | (15256)  Cyt B | 1 |
| 70 | 2/5 | TTC>CGA | (15258-15260)  Cyt B | 3 |
| 71 | 2/5 | TTA>CAG | (15262-15264)  Cyt B | 3 |
| 72 | 2/5 | ATTGGT>TCATTC | (15267-15272)  Cyt B | 6 |
| 73 | 2/5 | A>G | (15274)  Cyt B | 1 |
| 74 | 2/5 | AT>CC | (15276-15277)  Cyt B | 2 |
| 75 | 2/5 | GC>CT | (15279-15280)  Cyt B | 2 |
| 76 | 2/5 | T>A | (15282)  Cyt B | 1 |
| 77 | 2/5 | TAGCC>CCAAA | (15284-15288)  Cyt B | 5 |
| 78 | 2/5 | T>C | (15290)  Cyt B | 1 |
| 79 | 2/5 | AC>TA | (15292-15293)  Cyt B | 2 |
| 80 | 2/5 | T>G | (15298)  Cyt B | 1 |
| 81 | 2/5 | AT>TC | (15300-15301)  Cyt B | 2 |
| 82 | 2/5 | T>C | (15303)  Cyt B | 1 |
| 83 | 2/4 | CTA>GAT | (15413-15415) tRNA-Thr | 3 |
| 84 | 2/4 | AACT>GGAA | (15417-15420) tRNA-Thr | 4 |
| 85 | 2/4 | G>T | (15445)  tRNA-Pro | 1 |
| TOTAL |  |  |  | 299 |

Heteroplasmy of Cc2 turtle:

| Heteroplasmy | Coverage on reference sequence | Nucleotide change | position | length |
| --- | --- | --- | --- | --- |
| 1 | 2/5 | C>T | (3551)  ND 1 | 1 |
| 2 | 3/6 | GG>AA | (3764-3765) tRNA-Ile | 2 |
| 3 | 3/6 | C>A | (3767)  tRNA-Ile | 1 |
| 4 | 3/6 | CGTGCC>AAAAAA | (3769-3774) tRNA-Ile | 6 |
| 5 | 2/5 | T>A | (3934)  tRNA-Met | 1 |
| 6 | 2/5 | C>T | (3936)  tRNA-Met | 1 |
| 7 | 2/5 | CCGAA>AAACC | (3938-3942)  tRNA-Met | 5 |
| 8 | 2/5 | TGT>ACG | (3945-3947)  tRNA-Met | 3 |
| 9 | 2/5 | GGTT>CACA | (3949-3952)  tRNA-Met | 4 |
| 10 | 2/5 | A>C | (3954)  tRNA-Met | 1 |
| 11 | 2/5 | ATCCCTCCT>CGATACAAA | (3956-3964)  tRNA-Met | 9 |
| 12 | 2/5 | TACT>GGAG | (3966-3969) tRNA-Met | 4 |
| 13 | 2/5 | AT>CA | (3971-3972)  ND 2 | 2 |
| 14 | 2/5 | A>G | (3975)  ND 2 | 1 |
| 15 | 2/5 | CC>AA | (3979-3980)  ND 2 | 2 |
| 16 | 2/5 | GCTAA>CGATC | (5373-5377) tRNA-Tyr | 5 |
| 17 | 2/5 | ACACT>CGGGG | (5379-5383) tRNA-Tyr | 5 |
| 18 | 2/5 | GCCA>TGAG | (5386-5389) tRNA-Tyr | 4 |
| 19 | 2/5 | CCTA>AGGC | (5391-5394) tRNA-Tyr | 4 |
| 20 | 2/4 | A>G | (7776)  tRNA-Lys | 1 |
| 21 | 2/4 | A>G | (7781)  tRNA-Lys | 1 |
| 22 | 2/6 | T>C | (8012)  ATP 6 | 1 |
| 23 | 2/6 | A>C | (8780)  COX 3 | 1 |
| 24 | 2/6 | C>A | (8799)  COX 3 | 1 |
| 25 | 2/7 | A>C | (9471)  tRNA-Gly | 1 |
| 26 | 2/5 | AGCC>TTTT | (11309-11312)  ND 4 | 4 |
| 27 | 2/5 | A>T | (11314)  ND 4 | 1 |
| 28 | 2/5 | GA>TT | (11316-11317)  ND 4 | 2 |
| 29 | 2/5 | GAC>TTT | (11319-11321)  ND 4 | 3 |
| 30 | 2/5 | AC>TT | (11323-11324)  ND 4 | 2 |
| 31 | 2/5 | GC>TT | (11327-11328)  ND 4 | 2 |
| 32 | 2/5 | T>G | (11332)  ND 4 | 1 |
| 33 | 2/5 | A>T | (11335)  ND 4 | 1 |
| 34 | 2/5 | AAC>TGT | (11339-11341)  ND 4 | 3 |
| 35 | 2/5 | TAGCCC>GTTGAA | (11343-11348)  ND 4 | 6 |
| 36 | 2/5 | T>A | (11350)  ND 4 | 1 |
| 37 | 2/5 | C>A | (11352)  ND 4 | 1 |
| 38 | 2/5 | CAACCA>GGTGGT | (11355-11360)  ND 4 | 6 |
| 39 | 2/5 | AA>TT | (11363-11364)  ND 4 | 2 |
| 40 | 2/5 | CT>AA | (11366-11367)  ND 4 | 2 |
| 41 | 2/5 | AT>TC | (11369-11370)  ND 4 | 2 |
| 42 | 2/5 | GGA>TTG | (11372-11374)  ND 4 | 3 |
| 43 | 2/5 | AA>TC | (11376-11377)  ND 4 | 2 |
| 44 | 2/5 | AACC>GGTT | (11380-11383)  ND 4 | 4 |
| 45 | 2/5 | TTA>GAG | (11385-11387)  ND 4 | 3 |
| 46 | 2/5 | TACTT>CCAGG | (11389-11393)  ND 4 | 5 |
| 47 | 2/5 | CT>AG | (11396-11397)  ND 4 | 2 |
| 48 | 2/5 | T>A | (11399)  ND 4 | 1 |
| 49 | 2/5 | CC>GT | (11409-11410)  ND 4 | 2 |
| 50 | 2/5 | AC>TT | (11412-11413)  ND 4 | 2 |
| 51 | 2/5 | A>G | (11417)  ND 4 | 1 |
| 52 | 2/5 | A>T | (11420)  ND 4 | 1 |
| 53 | 2/5 | CC>TT | (11422-11423)  ND 4 | 2 |
| 54 | 2/5 | A>T | (11426)  ND 4 | 1 |
| 55 | 2/5 | AACAGGA>TGGGTTT | (11428-11434)  ND 4 | 7 |
| 56 | 2/5 | TAGGAACCC>GTTTTGTTT | (11436-11444)  ND 4 | 9 |
| 57 | 2/5 | AATC>TTGG | (11446-11449)  ND 4 | 4 |
| 58 | 2/5 | CC>TG | (11451-11452)  ND 4 | 2 |
| 59 | 2/5 | C>T | (11454)  ND 4 | 1 |
| 60 | 2/5 | AC>GG | (11456-11457)  ND 4 | 2 |
| 61 | 2/5 | TACA>GTGT | (11459-11462)  ND 4 | 4 |
| 62 | 2/5 | CCTAT>TTGTA | (11464-11468) ND 4 | 5 |
| 63 | 2/5 | ATA>CCG | (11471-11473)  ND 4 | 3 |
| 64 | 2/5 | TAT>CCC | (11475-11477)  ND 4 | 3 |
| 65 | 2/5 | TACAACACA>ATATTATGG | (11479-11487)  ND 4 | 9 |
| 66 | 2/5 | TGAGGGGAAACACC>  GTTTTAAGTTTGGT | (11489-11502)  ND 4 | 14 |
| 67 | 2/5 | TCATACATCAAAAC> CTTGGGGCGGCTTT | (11504-11517)  ND 4 | 14 |
| 68 | 2/5 | ATCCCCCCAACCCACACAC> GGTTGTGGGTTGTTAGTTA | (11519-11537)  ND 4 | 19 |
| 69 | 2/5 | AGA>TAT | (11539-11541)  ND 4 | 3 |
| 70 | 2/5 | CATCTCTTAA>  GGGGGGTCTT | (11543-11552)  ND 4 | 10 |
| 71 | 2/5 | ATCA>TGTT | (11554-11557)  ND 4 | 4 |
| 72 | 2/5 | TACACA>CTGTAG | (11559-11564)  ND 4 | 6 |
| 73 | 2/5 | CC>AT | (11566-11567)  ND 4 | 2 |
| 74 | 2/5 | ACCAA>GTTTT | (11569-11573)  ND 4 | 5 |
| 75 | 2/5 | C>T | (11582)  ND 4 | 1 |
| 76 | 2/5 | A>T | (11584)  ND 4 | 1 |
| 77 | 2/5 | TAACAA>GGTGGC | (11586-11591)  ND 4 | 6 |
| 78 | 2/5 | ACCAGAACTAA>  TATTAGTTATT | (11593-11603)  ND 4 | 11 |
| 79 | 2/5 | C>T | (11605)  ND 4 | 1 |
| 80 | 2/5 | AGGC>CTTA | (11608-11611)  ND 4 | 4 |
| 81 | 2/5 | C>T | (11613)  ND 4 | 1 |
| 82 | 2/5 | T>A | (11615)  ND 4 | 1 |
| 83 | 2/5 | A>G | (11619)  ND 4 | 1 |
| 84 | 2/5 | AA>GG | (11625-11626) tRNA-His | 2 |
| 85 | 2/5 | AT>GG | (11628-11629) tRNA-His | 2 |
| 86 | 2/5 | GTTT>AGAG | (11631-11634) tRNA-His | 4 |
| 87 | 2/5 | AAA>CTT | (11636-11638) tRNA-His | 3 |
| 88 | 2/5 | CAAACA>TCCTGT | (11640-11645) tRNA-His | 6 |
| 89 | 2/5 | TAGACT>GCTTGA | (11647-11652) tRNA-His | 6 |
| 90 | 2/5 | TGGC>GTAA | (11654-11657) tRNA-His | 4 |
| 91 | 2/5 | T>G | (11660)  tRNA-His | 1 |
| 92 | 2/5 | AAAATAGG>GGTGGGAT | (11662-11669) tRNA-His | 8 |
| 93 | 2/5 | G>A | (11671)  tRNA-His | 1 |
| 94 | 2/5 | A>T | (11674)  tRNA-His | 1 |
| 95 | 2/5 | AA>TG | (11676-11677) tRNA-His | 2 |
| 96 | 2/5 | CTCCTTA>GGTGAGT | (11679-11685) tRNA-His | 7 |
| 97 | 2/5 | A>T | (11688)  tRNA-His | 1 |
| 98 | 2/5 | CCGAGAGAGG>  TTTTAGTTCT | (11690-11699) tRNA-His; tRNA-Ser | 10 |
| 99 | 2/5 | A>G | (11701)  tRNA-Ser | 1 |
| 100 | 2/5 | AATAC>GGGGT | (11703-11707) tRNA-Ser | 6 |
| 101 | 2/5 | ATAA>TGGG | (11709-11712) tRNA-Ser | 4 |
| 102 | 2/5 | A>C | (11714)  tRNA-Ser | 1 |
| 103 | 2/5 | C>A | (11716)  tRNA-Ser | 1 |
| 104 | 2/5 | CTAAC>TGTTA | (11723)  tRNA-Ser | 5 |
| 105 | 2/5 | CCTA>TGAC | (11725-11728) tRNA-Ser | 4 |
| 106 | 2/5 | A>G | (11730)  tRNA-Ser | 1 |
| 107 | 2/5 | CTGAG>GGAGT | (11732-11736) tRNA-Ser | 5 |
| 108 | 2/5 | TA>GG | (11739-11740) tRNA-Ser | 6 |
| 109 | 2/5 | TCCC>AGGG | (11742-11745) tRNA-Ser | 4 |
| 110 | 2/5 | CA>AT | (11747-11748) tRNA-Ser | 2 |
| 111 | 2/5 | C>G | (11750)  tRNA-Ser | 1 |
| 112 | 2/5 | CCCTCACTTT>AGTGTTACAA | (11752-11761) tRNA-Ser; tRNA-Leu | 10 |
| 113 | 2/5 | AA>TG | (11763-11764) tRNA-Leu | 2 |
| 114 | 2/5 | G>T | (11766)  tRNA-Leu | 1 |
| 115 | 2/5 | A>C | (11768)  tRNA-Leu | 1 |
| 116 | 2/5 | A>G | (11770)  tRNA-Leu | 1 |
| 117 | 2/5 | AA>TG | (11772-11773) tRNA-Leu | 2 |
| 118 | 2/5 | TAATCCAC>GTTGTTTG | (11775-11782) tRNA-Leu | 8 |
| 119 | 2/5 | G>T | (11784)  tRNA-Leu | 1 |
| 120 | 2/5 | TT>CG | (11788-11789) tRNA-Leu | 2 |
| 121 | 2/5 | GG>TT | (11791-11792) tRNA-Leu | 2 |
| 122 | 2/5 | ACCACG>GATTTT | (11794-11799) tRNA-Leu | 6 |
| 123 | 2/8 | ACCCTTG>GTGGGGT | (11801-11807) tRNA-Leu | 7 |
| 124 | 2/8 | TGCAAT>GCATGG | (11809-11814) tRNA-Leu | 6 |
| 125 | 2/8 | CCA>TTT | (11816-11818) tRNA-Leu | 3 |
| 126 | 2/7 | G>T | (11820)  tRNA-Leu | 1 |
| 127 | 2/7 | AAAGTAAT>TTTTGGCA | (11823-11830) tRNA-Leu; ND 5 | 8 |
| 128 | 2/7 | ACCACAC>TTGGTTG | (11832-11838)  ND 5 | 7 |
| 129 | 2/7 | AA>TT | (11840-11841)  ND 5 | 2 |
| 130 | 2/7 | A>T | (11843)  ND 5 | 1 |
| 131 | 2/8 | CA>GC | (11848-11849)  ND 5 | 2 |
| 132 | 2/8 | CC>GG | (11851-11852)  ND 5 | 2 |
| 133 | 2/8 | CCTCT>GAGGG | (11853-11859)  ND 5 | 5 |
| 134 | 2/7 | AGCCC>TATTG | (11861-11865)  ND 5 | 5 |
| 135 | 2/7 | AAT>TGA | (11867-11869)  ND 5 | 3 |
| 136 | 2/7 | ACCC>GTTA | (11871-11874)  ND 5 | 4 |
| 137 | 2/7 | AATATT>TTGTTG | (11876-11881)  ND 5 | 6 |
| 138 | 2/7 | CCACT>GATGG | (11883-11887)  ND 5 | 5 |
| 139 | 2/7 | ACAACAA>GTTGGTT | (11889-11895)  ND 5 | 7 |
| 140 | 2/7 | T>G | (11897)  ND 5 | 1 |
| 141 | 2/7 | CCCA>GTTT | (11900-11903)  ND 5 | 4 |
| 142 | 2/7 | AAA>TCT | (11907)  ND 5 | 3 |
| 143 | 2/7 | A>G | (11909)  ND 5 | 1 |
| 144 | 2/7 | AACAC>GGGTA | (11912-11916)  ND 5 | 5 |
| 145 | 2/7 | ATAAAAACAA>TCGTCCGATT | (11919-11928)  ND 5 | 10 |
| 146 | 2/7 | AACAGCTGT>GGTGATGAG | (11930-11938)  ND 5 | 9 |
| 147 | 2/7 | AA>GT | (11941-11942)  ND 5 | 2 |
| 148 | 2/7 | TAGCAT>GTAATG | (11944-11949)  ND 5 | 6 |
| 149 | 2/7 | CT>TA | (11951-11952)  ND 5 | 2 |
| 150 | 2/7 | CAT>TGA | (11954-11956)  ND 5 | 3 |
| 151 | 2/7 | CCC>GGA | (11959-11961)  ND 5 | 3 |
| 152 | 2/7 | A>G | (11963)  ND 5 | 1 |
| 153 | 2/7 | TCCC>ATGA | (11965-11968)  ND 5 | 4 |
| 154 | 2/7 | C>G | (11970)  ND 5 | 1 |
| 155 | 2/7 | A>T | (11972)  ND 5 | 1 |
| 156 | 2/7 | CGCCT>ATAGA | (11975-11979) ND 5 | 5 |
| 157 | 2/7 | CA>TT | (11981-11982)  ND 5 | 2 |
| 158 | 2/7 | TTAT>AATG | (11984-11987)  ND 5 | 4 |
| 159 | 2/7 | CAGACA>GGCCTT | (11989-11994)  ND 5 | 6 |
| 160 | 2/7 | GAATC>TGTGA | (11997-12001)  ND 5 | 5 |
| 161 | 2/7 | TTATC>CGGAT | (12004-12008)  ND 5 | 5 |
| 162 | 2/7 | CCAACC>AAGTTG | (12010-12015)  ND 5 | 6 |
| 163 | 2/7 | CACT>ATTG | (12018-12021)  ND 5 | 4 |
| 164 | 2/7 | CA>AG | (12025-12026)  ND 5 | 2 |
| 165 | 2/7 | CCACATC>TTTGTGA | (12028-12034)  ND 5 | 7 |
| 166 | 2/7 | CACAT>TTTGC | (12035-12039)  ND 5 | 5 |
| 167 | 2/7 | C>T | (12041)  ND 5 | 1 |
| 168 | 2/7 | CCATAAACAT>GTCCTTTTGG | (12043-12052)  ND 5 | 10 |
| 169 | 3/6 | AGCTTTAAACTTG> CCAACGTTTTCAT | (12054-12066)  ND 5 | 13 |
| 170 | 3/6 | CA>TC | (12068-12069)  ND 5 | 2 |
| 171 | 2/7 | GT>GG | (12071-12072)  ND 5 | 2 |
| 172 | 2/7 | CTCCATCA>ACCTACAC | (12074-12081)  ND 5 | 8 |
| 173 | 2/7 | GTTC>TAGA | (12083-12086)  ND 5 | 4 |
| 174 | 2/7 | GTCCCAATCGCC> AAGTTGCGATGT | (12087-12098)  ND 5 | 12 |
| 175 | 2/5 | ATA>TGG | (12106-12108)  ND 5 | 3 |
| 176 | 2/5 | GTC>TAA | (12105-12107)  ND 5 | 3 |
| 177 | 2/5 | CA>TT | (12109-12110)  ND 5 | 2 |
| 178 | 2/5 | G>T | (12112)  ND 5 | 1 |
| 179 | 2/5 | TC>AG | (12114-12115)  ND 5 | 2 |
| 180 | 2/5 | ATC>TCA | (12118-12119)  ND 5 | 3 |
| 181 | 2/5 | GAAT>TTGG | (12123-12126)  ND 5 | 4 |
| 182 | 2/5 | TACAC>AGTGA | (12128-12132)  ND 5 | 5 |
| 183 | 2/5 | T>G | (12135)  ND 5 | 1 |
| 184 | 2/5 | ATACA>TGTAT | (12137-12141)  ND 5 | 5 |
| 185 | 2/5 | CTAC>GGTG | (12145-12148)  ND 5 | 4 |
| 186 | 2/5 | GACCC>TGAAG | (12150-12154)  ND 5 | 5 |
| 187 | 2/5 | TATA>ATGC | (12156-12159)  ND 5 | 4 |
| 188 | 2/5 | CACA>AGTG | (12161-12164)  ND 5 | 4 |
| 189 | 2/5 | A>T | (12167)  ND 5 | 1 |
| 190 | 2/5 | TTTC>GAGA | (12170-12173)  ND 5 | 4 |
| 191 | 2/5 | A>T | (12176)  ND 5 | 1 |
| 192 | 2/5 | ACCTACTAATTTTCC> GTGGGTGTTGGAGGG | (12178-12192)  ND 5 | 15 |
| 193 | 2/5 | AG>TT | (12194-12195)  ND 5 | 2 |
| 194 | 2/5 | A>T | (12197)  ND 5 | 1 |
| 195 | 2/5 | CCA>GGT | (12199-12201)  ND 5 | 3 |
| 196 | 2/5 | AATAA>TTGTT | (12203-12207)  ND 5 | 5 |
| 197 | 2/5 | C>A | (12209)  ND 5 | 1 |
| 198 | 2/5 | G>T | (12213)  ND 5 | 1 |
| 199 | 2/5 | AAC>GTG | (12215-12217)  ND 5 | 3 |
| 200 | 2/5 | GCCAACAAC>TTAGTTCTA | (12219-12227)  ND 5 | 9 |
| 201 | 2/5 | TAT>GGC | (12229-12231)  ND 5 | 3 |
| 202 | 2/5 | TCA>AGT | (12233-12235)  ND 5 | 3 |
| 203 | 2/5 | T>G | (12237)  ND 5 | 1 |
| 204 | 2/5 | CTTTA>AGGCC | (12239-12243)  ND 5 | 5 |
| 205 | 2/5 | T>A | (12245)  ND 5 | 1 |
| 206 | 2/5 | C>A | (12248)  ND 5 | 1 |
| 207 | 2/5 | GAGA>TGTT | (12250-12253)  ND 5 | 4 |
| 208 | 2/5 | GGAG>CTGT | (12255-12258)  ND 5 | 4 |
| 209 | 2/5 | GAA>TGC | (12262-12264)  ND 5 | 3 |
| 210 | 2/5 | CAT>GTG | (12266-12268)  ND 5 | 3 |
| 211 | 2/5 | CC>TT | (12271-12272)  ND 5 | 2 |
| 212 | 2/5 | TCCTCT>AATGTA | (12274-12279)  ND 5 | 6 |
| 213 | 2/5 | AA>GT | (12281-12282)  ND 5 | 2 |
| 214 | 2/5 | C>G | (12284)  ND 5 | 1 |
| 215 | 2/5 | G>T | (12286)  ND 5 | 1 |
| 216 | 2/5 | GA>AG | (12289-12290)  ND 5 | 2 |
| 217 | 2/5 | GA>TG | (12292-12293)  ND 5 | 2 |
| 218 | 2/5 | CC>TG | (12295-12296)  ND 5 | 2 |
| 219 | 2/5 | CCGAACAGA>GGAGTTTTT | (12299-12307)  ND 5 | 9 |
| 220 | 2/5 | GCAAAC>ATGGTG | (12309-12314)  ND 5 | 6 |
| 221 | 2/5 | C>G | (12316)  ND 5 | 1 |
| 222 | 2/5 | CAGCCC>ATTTAG | (12319-12324)  ND 5 | 6 |
| 223 | 2/5 | AC>GA | (12326-12327)  ND 5 | 2 |
| 224 | 2/5 | GCCA>TGAG | (12330-12333)  ND 5 | 4 |
| 225 | 2/5 | T>A | (12335)  ND 5 | 1 |
| 226 | 2/5 | TTTACAAC>GCCTGCGA | (12337-12344)  ND 5 | 8 |
| 227 | 2/5 | TATCGGAGACA>  ATGCTTCCTAT | (12347-12357)  ND 5 | 11 |
| 228 | 2/5 | CG>GC | (12359-12360)  ND 5 | 2 |
| 229 | 2/5 | CTAATCCTCAGTATAGCC> GGCGGGTGATTGGGTTGA | (12363-12380)  ND 5 | 18 |
| 230 | 2/5 | G>A | (12382)  ND 5 | 1 |
| 231 | 2/5 | C>T | (12384)  ND 5 | 1 |
| 232 | 2/5 | CAATAAACC>TGGGTTGTT | (12388-12396)  ND 5 | 9 |
| 233 | 2/5 | AAACAC>TCGTTT | (12398-12403)  ND 5 | 6 |
| 234 | 2/5 | TG>GT | (12405-12406)  ND 5 | 2 |
| 235 | 2/5 | A>G | (12409)  ND 5 | 1 |
| 236 | 2/5 | C>G | (12411)  ND 5 | 1 |
| 237 | 2/5 | CCAACAAATCT>  GTGGAGGGGTA | (12413-12423)  ND 5 | 11 |
| 238 | 2/5 | TACCCACAC>CGTGGGTGT | (12425-12433)  ND 5 | 9 |
| 239 | 2/5 | AATC>CTGT | (12435-12438)  ND 5 | 4 |
| 240 | 2/5 | CACC>TGTA | (12440-12443)  ND 5 | 4 |
| 241 | 2/5 | C>G | (12445)  ND 5 | 1 |
| 242 | 2/5 | CT>AG | (12447-12448)  ND 5 | 2 |
| 243 | 2/5 | C>T | (12450)  ND 5 | 1 |
| 244 | 2/5 | TCC>AGG | (12452-12454)  ND 5 | 3 |
| 245 | 2/5 | CT>TG | (12456-12457)  ND 5 | 2 |
| 246 | 2/5 | CT>GA | (12459-12460)  ND 5 | 2 |
| 247 | 2/5 | G>A | (12462)  ND 5 | 1 |
| 248 | 2/5 | A>C | (12464)  ND 5 | 1 |
| 249 | 2/5 | TA>GT | (12466-12467)  ND 5 | 2 |
| 250 | 2/5 | TCC>AAT | (12469-12471)  ND 5 | 3 |
| 251 | 2/5 | AGCCGC>GCGGTG | (12473-12478)  ND 5 | 6 |
| 252 | 2/5 | CAG>TGA | (12481-12483)  ND 5 | 3 |
| 253 | 2/5 | AAAATCAGCCCAA> GTTGCGGTTAGTG | (12485-12497)  ND 5 | 13 |
| 254 | 2/5 | C>A | (12500)  ND 5 | 1 |
| 255 | 2/5 | CC>AG | (12503-12504)  ND 5 | 2 |
| 256 | 2/5 | CCAC>AGGG | (12506-12509)  ND 5 | 4 |
| 257 | 2/5 | C>T | (12511)  ND 5 | 1 |
| 258 | 2/5 | T>A | (12513)  ND 5 | 1 |
| 259 | 2/5 | A>G | (12515)  ND 5 | 1 |
| 260 | 2/5 | TACC>GTTT | (12517-12520)  ND 5 | 4 |
| 261 | 2/5 | GCAGCT>TGTATG | (12522-12527)  ND 5 | 6 |
| 262 | 2/5 | AG>GT | (12530-12531)  ND 5 | 2 |
| 263 | 2/5 | AGGCCCCACC>  TTTATGGTTT | (12533-12542)  ND 5 | 10 |
| 264 | 2/5 | C>G | (12544)  ND 5 | 1 |
| 265 | 2/5 | GTT>TAA | (12546-12548)  ND 5 | 3 |
| 266 | 2/5 | C>G | (12550)  ND 5 | 1 |
| 267 | 2/5 | GCAT>TGTC | (12551-12555)  ND 5 | 4 |
| 268 | 2/5 | ACTAC>TTAGA | (12557-12561)  ND 5 | 5 |
| 269 | 2/5 | CTC>TAG | (12563-12565)  ND 5 | 3 |
| 270 | 2/5 | G>T | (12568)  ND 5 | 1 |
| 271 | 2/5 | ACTA>CAGT | (12570-12573)  ND 5 | 4 |
| 272 | 2/5 | GT>TA | (12576-12577)  ND 5 | 2 |
| 273 | 2/5 | C>G | (12581)  ND 5 | 1 |
| 274 | 2/5 | CTG>TAT | (12584-12585)  ND 5 | 3 |
| 275 | 2/5 | AA>CC | (12587-12588)  ND 5 | 2 |
| 276 | 2/5 | C>G | (12590)  ND 5 | 1 |
| 277 | 2/5 | TCC>GAG | (12592-12594)  ND 5 | 3 |
| 278 | 2/5 | A>G | (12596)  ND 5 | 1 |
| 279 | 2/5 | AT>TG | (12600-12601)  ND 5 | 2 |
| 280 | 2/5 | CGAAT>TACCA | (12603-12607)  ND 5 | 5 |
| 281 | 2/5 | C>T | (12609)  ND 5 | 1 |
| 282 | 2/5 | CCCCAT>GTTAGG | (12611-12616)  ND 5 | 6 |
| 283 | 2/5 | C>A | (12618)  ND 5 | 1 |
| 284 | 2/5 | A>G | (12620)  ND 5 | 1 |
| 285 | 2/5 | CC>GG | (12622-12623)  ND 5 | 2 |
| 286 | 2/5 | CCA>GGT | (12625-12627)  ND 5 | 3 |
| 287 | 2/5 | CAACAC>ATTGGT | (12629-12634)  ND 5 | 6 |
| 288 | 2/5 | CCC>GGA | (12637-12639)  ND 5 | 3 |
| 289 | 2/5 | C>T | (12641)  ND 5 | 1 |
| 290 | 2/5 | CAACCTG>GTGTAGT | (12643-12649)  ND 5 | 7 |
| 291 | 2/5 | TTGCC>CCTAT | (12653-12657)  ND 5 | 5 |
| 292 | 2/5 | AGGA>TTTC | (12659-12662)  ND 5 | 4 |
| 293 | 2/5 | CCATCACCACA> AATATCTTGTT | (12664-12674)  ND 5 | 11 |
| 294 | 2/5 | TA>GT | (12676-12677)  ND 5 | 2 |
| 295 | 2/5 | T>A | (12679)  ND 5 | 1 |
| 296 | 2/5 | AC>TT | (12681-12682)  ND 5 | 2 |
| 297 | 2/5 | C>A | (12685)  ND 5 | 1 |
| 298 | 3/6 | T>A | (12688)  ND 5 | 1 |
| 299 | 2/5 | TGCGCCCTC>GAATAATGG | (12690-12698)  ND 5 | 9 |
| 300 | 2/5 | C>A | (12700)  ND 5 | 1 |
| 301 | 2/5 | AA>TG | (12703-12704)  ND 5 | 2 |
| 302 | 2/5 | ATGAT>GCATA | (12706-12710)  ND 5 | 5 |
| 303 | 2/5 | TC>GA | (12712-12713)  ND 5 | 2 |
| 304 | 2/5 | A>T | (12715)  ND 5 | 1 |
| 305 | 2/5 | AA>GT | (12717-12718)  ND 5 | 2 |
| 306 | 2/5 | ATTA>TGGC | (12720-12723)  ND 5 | 4 |
| 307 | 2/5 | GCCTTCTCCACATC> TGAAGAATGCGTGT | (12726-12739)  ND 5 | 14 |
| 308 | 2/5 | A>T | (12741)  ND 5 | 1 |
| 309 | 2/5 | CCA>GAG | (12743-12745)  ND 5 | 3 |
| 310 | 2/5 | CTA>TAT | (12747-12749)  ND 5 | 3 |
| 311 | 2/5 | GCCTT>TAGGA | (12751-12755)  ND 5 | 5 |
| 312 | 2/5 | TAA>GGC | (12757-12759)  ND 5 | 3 |
| 313 | 2/8 | TGGCC>AAAAA | (14218-14222)  Cyt B | 5 |
| 314 | 2/8 | C>A | (14224)  Cyt B | 1 |
| 315 | 2/8 | CCT>AAA | (14227-14230)  Cyt B | 3 |
| 316 | 2/8 | CG>AA | (14232-14233)  Cyt B | 2 |
| 317 | 2/7 | A>C | (14849)  Cyt B | 1 |
| 318 | 2/5 | A>T | (15369)  tRNA-Thr | 1 |
| 319 | 2/7 | T>C | (16205)  [D-loop] | 1 |
| 320 | 3/7 | CC>AG | (16211-16212)  [D-loop] | 2 |
| 321 | 2/7 | TAC>CGG | (16215-16217)  [D-loop] | 3 |
| 322 | 3/7 | CC>GA | (16220-16221)  [D-loop] | 2 |
| 323 | 2/7 | A>C | (16215-16217)  [D-loop] | 1 |
| 324 | 2/5 | CC>AT | (16239-16240)  [D-loop] | 2 |
| 325 | 2/5 | CCAC>GAGA | (16243-16246)  [D-loop] | 4 |
| 326 | 2/5 | CCCA>TAAC | (16248-16251)  [D-loop] | 4 |
| 327 | 2/5 | A>G | (16258)  [D-loop] | 1 |
| TOTAL |  |  |  | 1173 |

Heteroplasmy of Cc3 turtle:

| **Heteroplasmy** | **Coverage on reference sequence** | **Nucleotide change** | **Position** | **Length** |
| --- | --- | --- | --- | --- |
| 1 | 2/4 | GA>TG | (32-33)  tRNA-Phe | 2 |
| 2 | 3/6 | A>C | (3881)  tRNA-Gln | 1 |
| 3 | 3/6 | T>A | (3889)  tRNA-Gln | 1 |
| 4 | 2/5 | AAA>GCC | (5354-5355) tRNA-Tyr | 3 |
| 5 | 2/5 | GGGACTACAGC> CTATTCCTGCT | (5357-5367) tRNA-Tyr | 11 |
| 6 | 2/5 | CAA>ATG | (5369-5371) tRNA-Tyr | 3 |
| 7 | 2/5 | G>C | (5373)  tRNA-Tyr | 1 |
| 8 | 2/5 | TA>CG | (5375-5376) tRNA-Tyr | 2 |
| 9 | 2/5 | TACACTCAGCC> AGATTAGGTAT | (5378-5388) tRNA-Tyr | 11 |
| 10 | 2/5 | CCC>GGG | (5390-5392) tRNA-Tyr | 3 |
| 11 | 2/5 | A>G | (5394)  tRNA-Tyr | 1 |
| 12 | 2/7 | C>G | (6694)  COX 1 | 1 |
| 13 | 2/7 | TGGAATACCA> GTCGTGGTAT | (6699-6708)  COX 1 | 10 |
| 14 | 3/6 | GCTCCCCT> AAAAAAAA | (9463-9470) tRNA-Gly | 8 |
| 15 | 3/6 | GT>AA | (9472-9473) tRNA-Gly | 2 |
| 16 | 3/6 | T>A | (9475)  tRNA-Gly | 1 |
| TOTAL |  |  |  | 61 |
